# Inhibition of HIV-1 replication by small interfering RNAs directed against Glioma Pathogenesis Related Protein (GliPR) expression

**DOI:** 10.1186/1742-4690-7-26

**Published:** 2010-03-31

**Authors:** Gianni Capalbo, Thea Müller-Kuller, Ursula Dietrich, Dieter Hoelzer, Oliver G Ottmann, Urban J Scheuring

**Affiliations:** 1 Department of Hematology/Oncology and Infectious Diseases, J. W. Goethe-University Hospital, Theodor Stern Kai 7, 60590 Frankfurt/Main, Germany; 2 Georg-Speyer-Haus, Institute for Biomedical Research, Paul-Ehrlich-Str. 42-44, 60596 Frankfurt/Main, Germany

## Abstract

**Background:**

Previously, we showed that glioma pathogenesis related protein (GliPR) is induced in CEM T cells upon HIV-1 infection *in vitro*. To examine whether GliPR plays a role as HIV dependency factor (HDF), we tested the effect of GliPR suppression by siRNA on HIV-1 replication.

**Results:**

Induction of GliPR expression by HIV-1 was confirmed in P4-CCR5 cells. When GliPR was suppressed by siRNA, HIV-1 replication was significantly reduced as measured by HIV-1 transcript levels, HIV-1 p24 protein levels, and HIV-1 LTR-driven reporter gene expression, suggesting that GliPR is a cellular co-factor of HIV-1. Microarray analysis of uninfected HeLa cells following knockdown of GliPR revealed, among a multitude of gene expression alterations, a down-regulation of syndecan-1, syndecan-2, protein kinase C alpha (PRKCA), the catalytic subunit β of cAMP-dependent protein kinase (PRKACB), nuclear receptor co-activator 3 (NCOA3), and cell surface protein CD59 (protectin), all genes having relevance for HIV-1 pathology.

**Conclusions:**

The up-regulation of GliPR by HIV-1 and the early significant inhibition of HIV-1 replication mediated by knockdown of GliPR reveal GliPR as an important HIV-1 dependency factor (HDF), which may be exploited for HIV-1 inhibition.

## Background

The replication of HIV-1 depends on specific host factors [1-4]. A recent report identified 273 cellular HIV-1 dependency factors (HDF), that are important for HIV-1 replication [5]. Furthermore, HIV-1 modifies the mRNA expression of a relatively large number of host cell genes, as shown by several reports [6-10]. Differential display experiments suggested that the expression of ~700 host genes (approximately 3% of all cellular genes) is modified by HIV-1 infection *in vitro *[9]. A microarray analysis using a limited subset of 1500 cDNAs identified 20 differentially expressed mRNAs from several cellular pathways [7]. Specific HIV-1 proteins including Tat, Nef, gp120 and Vpr were examined to dissect their role in modifying the transcription of cellular genes [11-14]. While some of the differentially expressed cellular genes may play a role in host defense mechanisms, others may facilitate HIV-1 replication, infectivity, species propagation and survival. A subgroup of differential cellular gene expressions may even support both host defense and viral replication, since HIV-1 replication is linked to immune activation of CD4+ T cells. Due to evolutionary selection, HIV-1 is expected to induce specific host factors, favorable for viral replication or propagation, and to suppress unfavorable cellular gene products [15-17]. Therefore, the examination of host cell genes, that are up-regulated upon HIV-1 infection, is expected to identify potential targets for inhibition of HIV-1 replication.

Previously, we found an early up-regulation of GliPR expression by more than 5-fold in CEM T cells infected with HIV-1 by a differential display screen [9]. Therefore, we were interested in delineating the role of GliPR for HIV-1 replication.

GliPR was identified originally in human glioblastomas [18] and was also described as *related to testes-specific, vespid, and pathogenesis protein 1 *(RTVP-1) [19]. Increased expression of GliPR was associated with myelomonocytic differentiation in macrophages [20]. Whereas GliPR has been reported to act as a tumor suppressor gene inducing apoptosis in prostate cancer [21-24], it appears to be an oncogene in glioblastomas [25] and Wilms tumors [26]. RTVP-1 protein was reported to contain a N-terminal signal peptide sequence and a transmembrane domain [27]. Furthermore, homology studies revealed a putative active enzymatic center in GliPR [27]. GliPR is homologous to group 1 plant pathogenesis-related proteins (PR-1) that are implicated in plant defense responses to viral, bacterial, and fungal infection [28,29]. Since GliPR shows structural similarities with its homologous plant PR-1 proteins, mammalian testis proteins (TPX1) and the insect venom Ag-5 protein, which are secretory proteins [29,30], it has been suspected that GliPR is also secreted. GliPR's homology with plant PR-1 proteins that have been attributed with a defense function may raise the question whether GliPR has an evolutionarily conserved role in innate immune response and human host defense of viral infection including HIV-1. Alternatively or additionally, HIV-1 may induce and exploit GliPR for viral replication.

The effect of GliPR knockdown on HIV-1 replication was studied, in order to test the hypotheses of GliPR being a host defense protein against or a co-factor of HIV-1. Furthermore, in order to identify downstream targets of GliPR, the effect of GliPR suppression on cellular gene expression was also investigated using cDNA microarrays.

## Results

### GliPR is induced upon HIV-1 infection in P4-CCR5 cells

**Figure 1:**
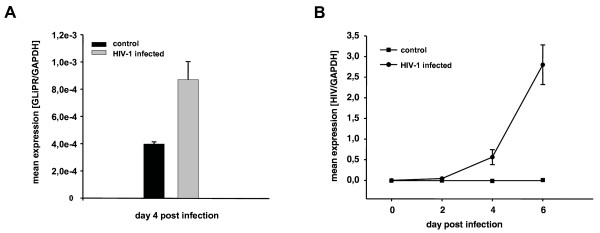
**Up-regulation of GliPR expression by HIV-1 infection**. **(A) **HIV-1_LAI_-infected P4-CCR5 cells and controls were subjected to quantitative PCR of GliPR expression at day 4 after HIV-1 infection. **(B) **In order to display HIV-1 infection kinetics, real-time quantitative PCR was also utilized to determine levels of intracellular HIV-1 viral mRNA normalized by cell number (house keeping gene GAPDH) in triplicate at day 0, 2, 4 and 6 post infection. Bars represent the standard deviation of the mean of determinations.

Since HIV-1 infection induced GliPR expression in HIV-1 infected human T cell line cells, as described previously [9], we tested whether this modification could be reproduced in P4-CCR5 HeLa cells infected with HIV-1_LAI_. P4-CCR5 HeLa cells were employed for the present study because they are more amenable to efficient transfection of synthetic siRNA compared to lymphocytic cell lines. Quantitative PCR demonstrated an up-regulation of GliPR transcripts by approximately 2-fold at day 4 after infection compared to uninfected cells (Fig. 1a). In order to display HIV-1 infection kinetics, real-time quantitative PCR was also utilized to determine levels of intracellular HIV-1 viral mRNA normalized by cell number (house keeping gene GAPDH) at different time points following infection (Fig. 1b). The data show that HIV-1 replication is still in the early logarithmic phase at day 4 in this cell culture system and that GliPR expression is induced in this early phase.

### Suppression of GliPR mediated by short interfering RNA

**Figure 2:**
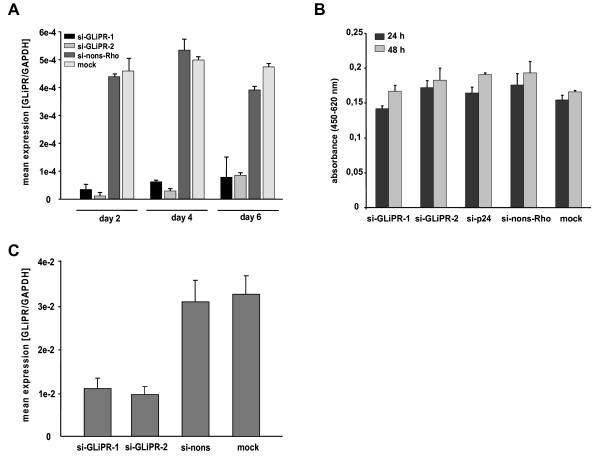
**Efficacy of siRNA-mediated suppression of GliPR**. **(A) **Quantitative PCR analysis of GliPR expression in P4-CCR5 cells which were transfected with 2 different siRNAs against GliPR or the control non-silencing siRNA labeled with rhodamine. Results are presented as mean values of triplicate samples ± standard deviation (SD). **(B) **Cell viability was determined with the WST-1 assay 24 h and 48 h after siRNA transfection. Results are expressed as absorbance (OD_450_). Bars represent the standard deviation of the mean of determinations. **(C) **Quantitative PCR analysis of GliPR expression in Jurkat cells 2 days after transfection with 2 different siRNAs against GliPR or the control non-silencing siRNA labeled with rhodamine.

P4-CCR5 cells were transfected with siRNAs specific for GliPR or a non-silencing siRNA, which was 5-prime labeled with rhodamine. Flow cytometry analysis of cells transfected with non-silencing siRNA 24 h post transfection revealed transfection efficiencies on average of 90% in all samples. Forty-eight hours after transfection, the relative levels of GliPR mRNA transcripts were decreased by at least 90%, as shown by quantitative real-time PCR (Fig. 2a). Even four and six days after transfection a markedly reduced GliPR expression by at least 80% compared with non-transfected cells (mock) or cells transfected with non-silencing siRNA was observed (Fig. 2a).

Viability and proliferation rate of P4-CCR5 cells transfected with siRNAs against GliPR or with the non-silencing siRNA remained unchanged as determined by WST-1 cell proliferation assay (Fig. 2b).

In order to establish a test system in a T cell line as well, a predominant type of host cell for HIV-1, Jurkat cells were transfected with 2 different siRNAs targeting GliPR, control non-silencing siRNA, or mock transfection without any siRNA. GliPR mRNA expression was reduced by around 64% to 69% at 48 hours after transfection with specific siRNAs compared to controls (Fig. 2c). The less pronounced reduction of GliPR expression compared to P4-CCR5 HeLa cells may be attributed to the lower transfection efficiency generally observed in T cell lines. In this experiment, approximately 70% of Jurkat cells were transfected, while 90% of P4-CCR5 HeLa cells were transfected.

In general, GliPR-directed siRNAs reduced the expression of GliPR effectively in P4-CCR5 and Jurkat cells without affecting cell viability.

### Down-regulation of GliPR by siRNA inhibits HIV-1 replication in P4-CCR5 and Jurkat cells

**Figure 3:**
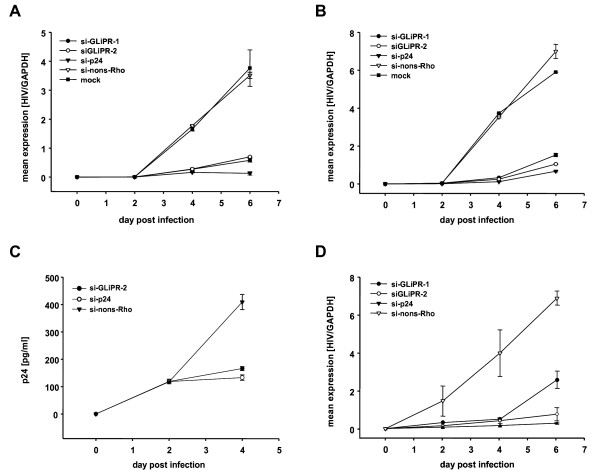
**Effects of siRNA transfections on HIV-1 replication**. P4-CCR5 cells were transfected with siRNAs directed against GliPR, viral p24 or an unspecific sequence (non-silencing control) and subsequently infected with HIV-1_LAI _with a multiplicity of infection of 0.01 **(A) **and 0.05 **(B)**, respectively. HIV-1 RNA copy numbers were normalized per cell count by house keeping gene GAPDH. **(C) **P4-CCR5 cells were transfected with siGliPR-2, si-p24 or non-silencing control siRNA and subsequently infected with HIV-1_LAI _with a multiplicity of infection of 0.01. Concentrations of viral p24 at day 0, 2 and 4 represent mean values of triplicate samples. **(D) **Jurkat cells were transfected with siRNAs directed against GliPR, viral p24 or an unspecific sequence (non-silencing control) and subsequently infected with HIV-1_LAI _with a multiplicity of infection 0.01. HIV-1 RNA copy numbers were normalized per cell count by house keeping gene GAPDH.

In order to examine the effect of GliPR knockdown on HIV-1 replication, P4-CCR5 cells were transfected with GliPR-specific siRNAs and subsequently infected with HIV-1_LAI_. As a negative control, the non-silencing siRNA (si-nons-Rho) was utilized while a siRNA targeting HIV-1 p24 was used as a positive control, since it was able to inhibit viral replication very effectively, as previously demonstrated [31]. HIV-1 infection was performed 24 h post siRNA transfection with a MOI of 0.01 or 0.05. Sequential cell-associated HIV-1 viral mRNA levels were determined by real-time quantitative PCR during 6 days after infection. As expected, the positive control siRNA (si-p24) exhibited a marked inhibition in viral mRNA transcription. Similarly, the siRNA-mediated reduction of GliPR expression was followed by significantly reduced viral mRNA transcript levels compared to HIV-1 infected controls, which were mock-transfected (mock) or transfected with the non-silencing siRNA (si-nons-Rho) at both MOI of 0.01 and 0.05 (Fig. 3a and 3b).

The effect of GliPR suppression on HIV-1 replication was confirmed by p24 ELISA, showing a significantly reduced p24 expression at day 4 post infection in cultures with GliPR knock-down compared to controls with non-silencing siRNA (Fig. 3c).

In order to test this phenomenon in T cells, Jurkat cells transfected with siRNAs specific to GliPR or control siRNA were infected with HIV-1 at a MOI of 0.01. GliPR-specific siRNAs resulted in a significant reduction of HIV-1 replication, similar to the positive control with siRNA against p24 (Fig 3d). Thus the T cell line results are in line with the data in P4-CCR5 cells.

**Figure 4:**
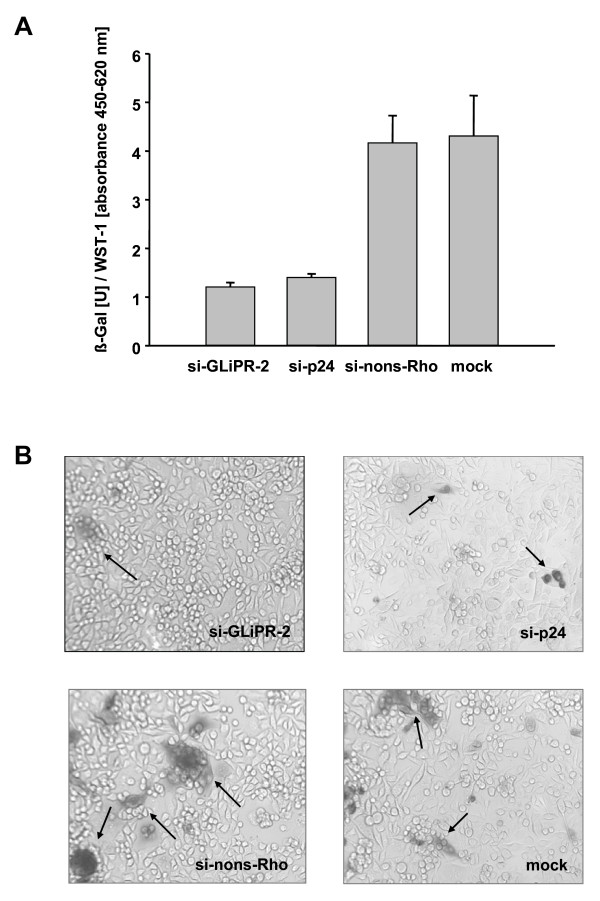
**Expression of β-galactosidase driven by HIV-1-LTR in HIV-infected cells after siRNA transfection**. P4-CCR5 cells containing a HIV-1-LTR driven β-galactosidase reporter vector were transfected with siGliPR-2, non-silencing control siRNA or no siRNA (mock) and subsequently infected with HIV-1_LAI _with a multiplicity of infection of 0.01. **(A) **β-galactosidase units at day 4 normalized by relative WST-1 values represent mean values from triplicate samples. **(B) **Photomicrograph of β-gal stained P4-CCR5 cells infected with HIV-1_LAI _(MOI 0.01) after transfection with mock, GliPR-siRNA, HIV-1 p24-siRNA or non-silencing control siRNA at day 6 post infection.

Furthermore, the effect on HIV-1 replication was examined by the integrated HIV-1-LTR-driven reporter vector expressing β-galactosidase in P4-CCR5 cells. HIV-1 Tat-mediated transactivation of the LTR leads to expression of measurable β-galactosidase activity, allowing measurments of inhibitory effects on HIV-1 replication as reductions in β-galactosidase activity. The expression of β-galactosidase was markedly decreased by siGliPR on day four after infection, comparable to the degree of the positive control with p24 siRNA (Fig. 4a). The inhibition of LTR-driven transcription was confirmed by microscopy of these cell cultures after X-Gal staining on day six after infection (Fig. 4b).

These results demonstrated that siRNA-mediated suppression of GliPR inhibited HIV-1 replication implicating that GliPR promotes HIV-1 replication. It was not possible to employ the opposite approach by assessing the effect of GliPR's over-expression on HIV-1 replication, since forced expression of GliPR caused rapid induction of apoptosis in HeLa cells and Jurkat cells (unpublished data).

### Differentially expressed genes after GliPR suppression with relevance for HIV-1 replication

In order to examine the effect of GliPR knock-down on cellular gene expression, microarray analyses were performed to identify cellular target genes of GliPR involved in HIV-1 pathology. The list of genes differentially expressed in uninfected HeLa cells following suppression of GliPR was screened for those genes which had been reported in the context of HIV-1 infection previously. Six genes with potential role in HIV-1 pathology were identified within the list of genes (n = 262) that were differentially regulated after GliPR suppression (Table 1).

**Table 1:** Differentially expressed genes in siGliPR transfected HeLa cells relevant to HIV-1 pathology.

Probe Set ID	Gene Title	Gene Symbol	fold change
201286_at	syndecan 1	SDC1	- 3.37
202741_at	protein kinase, cAMP-dependent, catalytic β	PRKACB	- 3.52
207700_at	nuclear receptor coactivator 3	NCOA3	- 6.53
212154_at 212157_at 212158_at	syndecan 2 (heparan sulfate proteoglycan)	SDC2	- 3.05 - 20.23 - 16.54
212463_at	CD59 antigen p18-20	CD59	-3.11
213093_at 215195_at	protein kinase C, alpha	PRKCA	- 3.55 - 4.09

Cellular genes that were found >3-fold induced (+) or suppressed (-) by siGliPR transfection of uninfected HeLa cells are listed with the factor of induction or suppression. Only genes that appeared to be relevant for HIV-1 infection according to a PubMed literature screen are listed.

## Discussion

The present investigation confirmed the up-regulation of GliPR induced by HIV-1 infection that we had found in a lymphocytic cell line [9] in P4-CCR5 HeLa cells. The suppression of GliPR expression by siRNA was associated with a significant inhibition of HIV-1 replication compared to controls as determined by quantitative PCR for HIV-1 transcripts, p24 ELISA, and HIV-1 LTR driven β-galactosidase expression. The inhibition of HIV-1 transcription following knockdown of GliPR was confirmed in Jurkat T cells. Furthermore, the knockdown of GliPR in uninfected HeLa cells revealed 6 differentially expressed genes, which had been reported to be associated with HIV-1 pathology.

The first hypothesis that GliPR induction is a cellular defense reaction hindering HIV-1 replication has to be questioned. If GliPR had exerted an anti-viral effect against HIV-1, the suppression of GliPR would have been expected to show an enhancing effect on HIV-1 replication, which was not found to be the case.

The second hypothesis that GliPR acts as a HIV-1 co-factor has to be favored, since down-regulation of GliPR expression caused a significant inhibition of HIV-1 replication, implying that GliPR promotes HIV-1 replication. This result could not be corroborated by the inverse technique of over-expressing GliPR because over-expression of GliPR caused apoptosis as reported for prostate cancer cell lines [22] and confirmed by us for HeLa and Jurkat cells (unpublished data). Apoptosis would have disguised a putative promoting effect on HIV-1 replication, since apoptosis per se would have affected the kinetics of HIV-1 replication.

The initiation of apoptosis in an infected cell may be considered to be an ancient defense mechanism aimed at the abortion of infection for the organism. Apparently, HIV-1 escapes this putative mechanism of defense as long as its replication has not been completed by blocking apoptosis initially via up-regulation of bcl-2 via Tat [32-34] or by other mechanisms, e.g. reduction of Bax or inhibition of ISG15 [35,36].

Moreover, HIV-1 appears to exploit a specific function of GliPR or GliPR-induced gene products for its replication. Therefore, GliPR may be considered as an HDF [1-4]. A recent report identified 237 HDF in a broad siRNA screen, in addition to 36 already described HDF [5]. GliPR was not found by this HDF screen, although our results suggest that it meets the criteria for an HDF. Apparently the detection of HDF depends on the specific experimental conditions and the selection criteria set for sensitivity and specificity of a screening approach.

It has been shown that tumor suppressor protein p53 is a direct transcriptional activator of GliPR. GliPR may also be induced independently of p53 [22]. It has been reported that HIV-1 induces or activates p53 [37,38], suggesting a p53-mediated pathway for up-regulation of GliPR by HIV-1.

It is also of interest to understand the mechanism by which GliPR promotes HIV-1 replication. The modeling of GliPR's protein structure revealed an active site [27]. Protein Tex31, which is a member of the PR-1 protein family and a component of the cone snail venom, is a substrate specific protease [39]. Golgi associated PR-1 related protein-1 (GAPR-1) also called GliPR2 due to its close homology with GliPR, is a putative serine protease, too [40]. It may be hypothesized that a potential GliPR protease activity may be involved in the processing of a specific HIV-1 protein as it has been demonstrated for other cellular proteases such as furin and PC7 in processing of gp160 [41]. The interaction of GliPR with HIV-1 polypeptides is possible since GliPR is localized in the endoplasmic reticulum (unpublished data).

Our microarray screen found 6 gene products down-regulated by GliPR suppression that have been reported in the context of HIV:

Syndecan-1 and syndecan-2 are heparansulfate proteoglycans and membrane proteins involved in cellular proliferation, migration and matrix interactions. Syndecan-1 functions as receptor for internalizing extracellular Tat protein [42] and syndecan-2 as regulator of T cell activation [43].

The catalytic subunit β of cAMP-dependent protein kinase (PRKACB) phosphorylates HIV-1 precursor protein and matrix protein p17 thereby initiating their translocation [44]. PRKACB is incorporated in HIV-1 virions and regulates viral infectivity [45]. PRKCA phosphorylates Tat and transactivates HIV-1-LTR [46].

The nuclear receptor coactivator 3 (NCOA3) interacts with Tat enhancing Tat's transactivating effect [47].

The cell surface protein CD59 (protectin) prevents the formation of membrane attack complexes (MAC). CD59 co-localizes with HIV-1 matrix protein p17 in virions [48].

## Conclusions

GliPR is induced in early HIV-1 infection *in vitro*. According to the profound inhibitory effect of GliPR knock-down on HIV-1 replication, GliPR is an important HDF. Future research needs to address whether GliPR directly functions as a co-factor of HIV-1 processing or whether it exerts its effect via other cellular target genes as identified by our microarray screen. It is also important to define the role of GliPR in cellular defense against other viral infections in general.

## Methods

### Cell culture and HIV-1 infection

For siRNA knock-down experiments, HeLa cells and P4-CCR5 [49] cells (HeLa CD4^+ ^CCR5^+ ^long terminal repeat-LacZ) were cultured in Dulbecco's modified Eagle's medium (Invitrogen, Karlsruhe, Germany). C8166 and Jurkat cells were cultured in RPMI 1640 medium (Invitrogen). All media were supplemented with 10% fetal calf serum (Gibco-BRL, Karlsruhe, Germany), 1% glutamine (Gibco-BRL) and 1% antibiotic solution (penicillin and streptomycin; Gibco-BRL). P4-CCR5 cells were cultured in the presence of 100 μg/ml G418 (PAA Laboratories, Coelbe, Germany) and 1 μg/ml Puromycin (PAA Laboratories). Transfection and infection of the cells were carried out in the absence of any antibiotics. The HIV-1 strain LAI was taken from the supernatant fluid of freshly infected H9 cells. Viral titer (TCID_50 _units/ml) was determined by titration on C8166 cells as described [50].

24 h after siRNA transfection, P4-CCR5 or Jurkat cells were infected with HIV-1_LAI _in triplicate at a multiplicity of infection (MOI) of 0.01 or 0.05. Infection of P4-CCR5 cells was carried out in the presence of 50 μg/ml DEAE-Dextran (Sigma, Taufkirchen, Germany). After incubation for 4 h the cells were washed with PBS and re-fed with fresh medium. Cells and supernatant samples were collected for quantitative PCR analysis, β-galactosidase enzyme assay and HIV-1 p24 antigen ELISA at indicated time points.

### siRNA transfection

siRNAs with the following sequences were used: a positive control siRNA against HIV-1 p24 [31], (si-p24: 5'-GAU UGU ACU GAG AGA CAG Gdtdt-3'); a negative control non-silencing siRNA with no known homology to mammalian genes (si-nons-Rho: 5'-UUC UCC GAA CGU GUC ACG Udtdt-3'; 5-prime labeled with rhodamine); and two different siRNAs specific to GliPR (si-GliPR-1: 5'-GGU GAA ACC AAC AGC CAG Udtdt-3'; si-GliPR-2: 5'-GGA CUA UGA CUU CAA GAC Udtdt-3'). All siRNAs were synthesized by Ambion (Darmstadt, Germany) and were purchased as annealed RNA-duplexes. 24 h before transfection, HeLa or P4-CCR5 cells were plated in 24-well plates (Corning, Kaiserslautern, Germany) at 5 × 10^4 ^cells per well in Dulbecco's minimal essential medium containing 10% FBS with no antibiotics. Transfections were performed with Lipofectamine 2000 transfection reagent (Invitrogen) with siRNA at a final concentration of 20 nM according to the manufacturer's recommendations. After incubating for 6 h, the lipid/siRNA complexes were removed and replaced with fresh medium. For further analysis, cells were removed from the culture dish by trypsinization with 0.25% trypsin/0.02% EDTA in PBS (Cambrex, Verviers, Belgium) at different time points after transfection. Transfection of Jurkat cells was performed with HiPerFect Transfection Reagent (Qiagen, Hilden, Germany) with siRNA at a final concentration of 100 nM according to the manufacturer's recommendations. Transfection efficiency was analyzed by flow cytometry 24 h after transfection. Data were acquired and analyzed on FACScan with Cell Quest software (Becton Dickinson, Heidelberg, Germany). Effects on cellular viability after siRNA treatment were measured using the cell proliferation reagent WST-1 according to the manufacturer's instructions (Roche, Penzberg, Germany).

### Real time PCR quantification of viral and cellular RNA

RNA was extracted using the RNeasy mini kit (Invitrogen) including treatment with RNase-free DNase I (Qiagen). Synthesis of cDNA was carried out using random hexamer primers and Superscript-II RNaseH-reverse transcriptase according to the manufacturer's specifications (Invitrogen).

Real-time PCR was performed in duplicate reactions employing ABI PRISM 7700 (Applied Biosystems, Darmstadt, Germany) with standard conditions (50°C for 2 min, 95°C for 10 min and 40 cycles at 95°C for 15 s and 60°C for 1 min). The 25 μl PCR included 2,5 μl cDNA, 1× TaqMan^® ^Universal PCR Master Mix (Applied Biosystems), 0.2 μM TaqMan^® ^probe, 0.2 μM forward primer and 0.2 μM reverse primer. Primers and probes were designed using Primer Express v.1.0 software (Applied Biosystems) and were synthesized by Thermohybaid (Ulm, Germany). In order to quantify GliPR, HIV-pol and GAPDH cDNA, the following primers and probes were used: GliPR (sense: 5'-TGC CAG ACA AAG CAT GCG T-3'; antisense: 5'-GCT GTG TGT GAA TAA TTG GAG ACA A-3'; probe: 5'-FAM-TCA CAC TTG CTA CAA TAG CCT GGA TGG TTT C-3'-TAMRA), HIV-*po*l (sense: 5'-AAT TTC ACC AGT ACT ACG GTT AAG GC-3'; antisense: 5'-CTT TAA TTC TTT ATT CAT AGA TTC TAC TAC TCC TTG-3'; probe: 5'-FAM-TGT TGG TGG GCG GGA ATC AAG C-3'-TAMRA) and GAPDH (sense: 5'-GAA GGT GAA GGT CGG AGT C-3'; antisense: 5'-GAA GAT GGT GAT GGG ATT TC-3'; probe: 5'-FAM-CAA GCT TCC CGT TCT CAG CC-3'-TAMRA). The probes were labeled with FAM at the 5' end and TAMRA at the 3' end. Copy numbers of the respective transcripts were calculated by plasmid standard curves, normalized by GAPDH housekeeping gene transcripts. Standard curves were obtained after amplification of log step dilutions between 10 to 10^6^copy numbers of purified plasmids carrying the amplicons of GliPR, HIV-1-pol (modified plasmid pLAI.2 obtained from the NIH AIDS Research & Reference Reagent Program) or human GAPDH [51], respectively. The plasmid standard for the quantification of GliPR was prepared by inserting a PCR-generated fragment (sense: 5'-GGA TCC ATG CGT GTC ACA CTT GCT ACA ATA GC-3' and antisense: 5'-GTC GAC TTA GTC CAA AAG AAC TAA ATT AGG GTA CTT GAG C-3') into pCR2.1 (Invitrogen), which was amplified using HeLa cDNA as template.

### β-Gal staining of cells

At indicated time points after HIV-1_LAI _infection, P4-CCR5 cells were washed twice with PBS and fixed for 5 min in fixative (0.25% glutaraldehyde in PBS) at room temperature. After two washes with PBS, cells were covered with staining solution (PBS containing 4 mM potassium ferrocyanide, 4 mM potassium ferricyanide, 2 mM MgCl_2_, and 0.4 mg/ml of X-Gal [5-bromo-4-chloro-3-indolyl-β-D-galactopyranoside]) and incubated at 37°C. Subsequently plates were washed twice with PBS and numbers of β-Gal-positive (blue) cells were examined microscopically.

### β-galactosidase enzyme assay

Cell lysates were prepared by using Reporter Lysis Buffer (Promega, Mannheim, Germany). To perform 96-well plate β-galactosidase assays 50 μl of cell lysates and 50 μl of 2× β-galactosidase assay buffer (Promega) were mixed and incubated at 37°C for 30 min. To stop the reaction, 150 μl of 1 M sodium carbonate was added to the mixture and mixed well by vortexing briefly. Absorbance of the reaction mixture was read immediately at 420 nm. A standard curve was created, using standards between 0 and 6.0 × 10^-3 ^units of Galactosidase (Promega). Reporter assay results were normalized according to relative cell numbers, which were estimated by using the cell proliferation reagent WST-1 according to the manufacturer's instructions (Roche).

### HIV-1 p24 antigen ELISA

HIV-1 p24 ELISA was performed using a commercially available kit (Beckmann Coulter, Krefeld, Germany) according to the manufacturer's instructions. For measuring p24 in the supernatants, 100-fold dilutions of the supernatants were used. All ELISA measurements were done in triplicate.

### Gene expression analysis by microarrays

The microarray analysis of the effect of GliPR suppression by siRNA was performed using the HG-U133 Plus 2.0 microarray of Affymetrix (Santa Clara, CA USA) per manufacturer's instructions (GeneChip^® ^Expression Analysis Manual). This chip contains 47,000 transcripts which represent 39,000 annotated genes. The data analysis was carried out according to established standards for Affymetric microarrays using *GeneChip Operating Software *(GCOS; Affymetrix) and *GeneSpring *(Agilent Technologies).

## Competing interests

The authors declare that they have no competing interests.

## Authors' contributions

GC performed the experiments and contributed to draft the manuscript. TMK contributed to the experiments and to the analysis of data. DH, UD and OGO participated in the design of the study and helped to draft the manuscript. UJS directed the work and completed the manuscript. All authors read and approved the final manuscript.
